# Diagnostic Accuracy of Prognostic Nutritional Index and Systemic Immune–Inflammatory Index in Predicting Fibrosis and Histological Activity in Chronic Hepatitis B

**DOI:** 10.3390/nu18091332

**Published:** 2026-04-23

**Authors:** Ali Can Uguz, Mehmet Bayram, Hafize Uzun, Omur Tabak

**Affiliations:** 1 Department of Internal Medicine, Kanuni Sultan Suleyman Training and Research Hospital, University of Health Sciences, 34303 Istanbul, Türkiye; alicanuguz1@hotmail.com; 2 Department of Gastroenterology, Türkiye Hospital, 34381 Istanbul, Türkiye; drmhbayram@gmail.com; 3 Department of Medical Biochemistry, Faculty of Medicine, Istanbul Atlas University, 34403 Istanbul, Türkiye; huzun59@hotmail.com

**Keywords:** prognostic nutritional index, systemic immune–inflammatory index, fibrosis, hepatitis B

## Abstract

**Background:** Liver biopsy remains the gold standard for staging chronic hepatitis B (CHB), yet it is invasive, costly, and associated with potential complications. There is a critical need for non-invasive, cost-effective biomarkers to monitor disease progression. This study aimed to evaluate the correlation between the Prognostic Nutritional Index (PNI) and Systemic Immune–Inflammatory Index (SII) with histological fibrosis stages and the Histological Activity Index (HAI) in patients with CHB. **Methods:** This retrospective study analyzed 274 patients diagnosed with CHB (HBsAg positivity > 6 months) who underwent liver biopsy at the University of Health Sciences, Kanuni Sultan Süleyman Training and Research Hospital between February 2016 and February 2022. Histopathological findings were staged using the Ishak fibrosis score and HAI. PNI and SII were calculated from peripheral blood parameters. Statistical discrimination power was assessed using Area Under the Receiver Operating Characteristic (AUROC) curves. **Results:** The cohort comprised 119 females (43.4%) and 155 males (56.6%), with a mean age of 45.25 ± 11.2 years. Mean values were 55.83 ± 5.33 for PNI and 494.37 ± 336.86 for SII. Fibrosis distribution showed 56.2% at stages F0–F1 and 43.8% at ≥F2. For fibrosis staging, SII demonstrated statistically significant but limited predictive ability for Ishak scores ≥F2, while PNI was significant for identifying advanced fibrosis (≥F4) (*p* < 0.05). SII showed moderate diagnostic performance for severe inflammation (HAI ≥12; AUROC = 0.848), although this finding should be interpreted cautiously. For lower HAI thresholds (≥6), both PNI and SII demonstrated poor discriminative ability (AUROC 0.5–0.6). **Conclusions:** Both indices were associated with histological parameters but showed limited overall diagnostic performance. SII appeared relatively better; however, this was descriptively observed without formal statistical comparison. These markers may provide complementary information but should not be used as standalone diagnostic tools.

## 1. Introduction

Hepatitis B virus (HBV) is a 42 nm hepadnavirus characterized by a partially double-stranded DNA genome, an inner core antigen (HBcAg), and a surface antigen (HBsAg). HBV remains a critical global health burden, capable of inducing acute and chronic hepatic inflammation, which may progress to end-stage complications such as cirrhosis and hepatocellular carcinoma (HCC) [1]. According to 2019 World Health Organization (WHO) data, approximately 296 million individuals live with chronic HBV infection (CHB), with 1.5 million new infections occurring annually. In the same year, HBV-related mortality reached 820,000, primarily due to cirrhosis and HCC [2]. In Turkey, the European Centre for Disease Prevention and Control (ECDC) estimates that 3 million people are infected, with HBsAg prevalence reaching 64% in cirrhotic patients and 54% in those with HCC [3].

The diagnosis, staging, and therapeutic management of CHB rely on a combination of biochemical markers (AST, ALT), serological profiles (HBsAg, HBeAg, anti-HBc IgM/IgG), and molecular quantification of HBV DNA. While these parameters categorize the disease phases, histopathological evaluation via liver biopsy remains the gold standard for assessing necroinflammation and fibrosis [4]. Several scoring systems are utilized for staging, including the Knodell Histological Activity Index (HAI), Metavir, and the Ishak modified score. The HAI provides a composite score (0–18) reflecting periportal necrosis, intralobular degeneration, and portal inflammation [5]. The Ishak system, a refinement of the Knodell score, offers higher sensitivity for detecting subtle fibrotic changes across six stages, making it a preferred tool in clinical trials [6].

Despite its diagnostic utility, liver biopsy is invasive and carries inherent risks of complications. Consequently, there is an increasing demand for non-invasive, cost-effective, and reproducible biomarkers to predict disease activity and prognosis. The Prognostic Nutritional Index (PNI) and the Systemic Immune–Inflammation Index (SII) have emerged as potent markers in oncological and geriatric contexts. PNI, calculated from serum albumin and absolute lymphocyte count, reflects both nutritional status and systemic inflammation; given that albumin is synthesized in the liver, low PNI scores often correlate with impaired hepatic function and poor prognosis [7]. Conversely, SII integrates platelet (P), neutrophil (N), and lymphocyte (L) counts [SII = (PXN)/L] to represent the balance between systemic inflammation and host immune response. Elevated SII levels typically indicate robust inflammation and a weakened immune profile [8].

This study aimed to evaluate the association between PNI and SII values and histopathological fibrosis and inflammatory activity in patients with CHB. We conducted a retrospective analysis of patients who underwent liver biopsy at a tertiary care center, using histopathological findings as the reference standard to assess the diagnostic performance and potential clinical relevance of these indices. In this context, we sought to explore whether these readily available, non-invasive parameters could provide complementary information in the evaluation of disease severity.

## 2. Materials and Methods

### 2.1. Ethical Considerations

The study protocol was reviewed and approved by the Academic Ethics Committee of the University of Health Sciences, Istanbul Kanuni Sultan Suleyman Training and Research Hospital (Date: 9 March 2022; Approval No: 2022/03/63). The study was conducted in strict accordance with the ethical principles of the Declaration of Helsinki. Since this was a retrospective chart review, the requirement for informed consent was waived by the ethics committee.

### 2.2. Study Design and Population

This retrospective cohort study included patients who applied to the Gastroenterology and Internal Medicine outpatient clinics at the University of Health Sciences, Kanuni Sultan Suleyman Training and Research Hospital, between February 2016 and February 2022.

#### 2.2.1. Inclusion Criteria

- Patients diagnosed with CHB, defined by HBsAg positivity for >6 months and HBV DNA levels > 2000 IU/mL.
- Availability of comprehensive laboratory data [Complete Blood Count (CBC), ALT, AST, serum albumin, and HBeAg (e-antigen) status].
- Documented liver biopsy results, including Ishak fibrosis stages and HAI scores.

#### 2.2.2. Exclusion Criteria

- Age < 18 years, pregnancy, or breastfeeding.
- Presence of HCC, other systemic malignancies, or benign hepatic masses.
- Co-infection with other viral hepatitides (HCV, HDV) or concomitant liver diseases (Autoimmune Hepatitis, Primary Biliary Cholangitis).
- Current use of immunosuppressive therapy (e.g., corticosteroids or azathioprine).
- Incomplete medical records or inadequate histopathological sampling.

Data were extracted from the hospital information system (PANATES). No additional tests were performed for the purpose of this study.

Missing data, including HBeAg status, were handled using a complete-case analysis approach, and no imputation methods were applied.

### 2.3. Clinical Indices

The primary non-invasive inflammatory and nutritional markers were calculated based on peripheral blood parameters obtained at the time of liver biopsy:

- PNI = 10 × serum albumin (g/dL) + 0.005 × total lymphocyte count (per m^3^)
- SII = (Platelet count × Neutrophil count)/Lymphocyte count

### 2.4. Histopathological Assessment

All liver biopsy specimens were evaluated by experienced pathologists blinded to the clinical and laboratory data. Histopathological findings were graded and staged using two standardized systems:

*Necro-inflammatory Activity:* Assessed via the Knodell HAI, a composite score (range 0–18) evaluating periportal necrosis, intralobular degeneration, and portal inflammation. For statistical analysis, patients were stratified by activity severity into groups of HAI < 6 (mild), HAI ≥ 6 (moderate), and HAI ≥ 12 (severe necroinflammation).

*Fibrosis Staging:* Evaluated using the Ishak modified scoring system (range 0–6). To determine the discriminative power of the PNI and SII, patients were categorized into clinically significant subgroups:

- Significant Fibrosis: Ishak score ≥ F2.
- Advanced Fibrosis/Cirrhosis: Ishak score ≥ F4.

The biopsy specimens were considered adequate for inclusion only if they contained a minimum of 6–10 portal tracts to ensure accurate staging and to minimize sampling error.

### 2.5. Statistical Analysis

Statistical analyses were performed using SPSS version 22.0 (IBM Corp., Armonk, NY, USA). Categorical variables are presented as frequencies and percentages, while continuous variables are reported as mean ± standard deviation (SD), median, and range. Data normality was assessed using the Kolmogorov–Smirnov test (n ≥ 50) or the Shapiro–Wilk test (n < 50). For continuous variables, independent groups were compared using Student’s *t*-test (parametric) or the Mann–Whitney U test (non-parametric). Categorical data were analyzed using the Pearson Chi-square test. The diagnostic performance and optimal cut-off values for PNI and SII in predicting HAI and fibrosis stages were determined using Receiver Operating Characteristic (ROC) curve analysis. The Area Under the Curve (AUC) was calculated to assess discriminative power, where an AUC < 0.5 indicates no diagnostic utility. Sensitivity, specificity, positive predictive value (PPV), negative predictive value (NPV), and the Youden Index were calculated for significant thresholds. A *p*-value < 0.05 was considered statistically significant.

## 3. Results

### 3.1. Patient Characteristics and Clinical Demographics

The study cohort consisted of 274 patients diagnosed with CHB. The demographic distribution included 155 males (56.6%) and 119 females (43.4%), with a mean age of 45.25 ± 16.25 years (range: 21–78). Serological evaluation revealed that 71.2% (n = 195) were HBeAg-negative, while 10.6% (n = 29) were HBeAg-positive (data were unavailable for 18.2%). The mean serum albumin level was 4.43 ± 0.35 g/dL, and the mean HBV DNA level was 7.6 × 10^7^ ± 2.9 × 10^8^ IU/mL. Regarding the primary non-invasive indices, the mean PNI and SII scores were 55.83 ± 5.34 and 494.37 ± 336.86, respectively.

### 3.2. Distribution of Clinical Indices and Histopathological Scores

The mean values for the non-invasive indices were 55.83 ± 5.34 for the PNI and 494.37 ± 336.87 for the SII. Upon histopathological examination of the liver biopsy specimens, the cohort exhibited a mean Ishak fibrosis stage of 1.51 ± 1.10 and a mean Knodell HAI score of 5.94 ± 2.34. The median values for fibrosis and HAI were 1.00 and 6.00, respectively, indicating a population primarily characterized by mild-to-moderate histological disease activity. The detailed descriptive statistics for these parameters are summarized in Table 1.

### 3.3. Histopathological Findings

Histopathological analysis using the Ishak scoring system revealed a mean fibrosis stage of 1.51 ± 1.10. The distribution of fibrosis was as follows: F0 (14.6%), F1 (41.6%), F2 (29.2%), F3 (9.5%), F4 (3.3%), F5 (1.1%), and F6 (0.7%). The mean Knodell HAI score was 5.94 ± 2.33. When stratified by necro-inflammatory activity, 65.3% (n = 179) of patients exhibited mild activity (HAI ≤ 6), while 34.7% (n = 95) showed moderate-to-severe activity (HAI > 6).

### 3.4. Comparative Analysis of Laboratory Parameters by Disease Severity

When patients were stratified by fibrosis severity (Ishak < 3 vs. ≥3), significant differences were observed in platelet counts (230.00 ± 66.68 vs. 199.90 ± 60.99 × 10^3^/µL; *p* = 0.010) and serum albumin levels (4.46 ± 0.32 vs. 4.27 ± 0.46 g/dL; *p* = 0.002). However, no significant differences were found for ALT, AST, neutrophil, or lymphocyte counts (*p* > 0.05).

Regarding necro-inflammatory activity (HAI ≤ 6 vs. > 6), moderate-to-severe activity was associated with significantly higher AST (*p* = 0.001) and ALT (*p* = 0.005) levels, and lower serum albumin (*p* = 0.03). No significant correlation was found between age, gender, or HBeAg status and the severity of fibrosis or HAI scores (*p* > 0.05).

### 3.5. Comparison of Biochemical and Hematological Parameters by HAI Score

The cohort was stratified based on the Knodell HAI to compare clinical parameters between different levels of necro-inflammatory activity. Patients were categorized into two groups: those with minimal-to-low activity (HAI ≤ 6, n = 179) and those with moderate-to-high activity (HAI > 6, n = 95).

Statistical analysis revealed that AST, ALT, and serum albumin levels differed significantly between the two groups (*p* < 0.05). Specifically, patients in the higher activity group (HAI > 6) exhibited significantly higher transaminase levels and lower albumin concentrations compared to the low-activity group. Conversely, no statistically significant differences were observed for neutrophil, lymphocyte, or platelet counts between the stratified HAI groups (*p* > 0.05), indicating that these individual hematological parameters lacked sufficient discriminative power regarding necro-inflammatory activity levels in this cohort.

### 3.6. Impact of Demographic Variables and HBeAg Status on Histopathological Outcomes

The study evaluated whether demographic characteristics and HBeAg serostatus influenced the severity of liver disease. Patients were stratified into two groups based on the Ishak fibrosis score: mild-to-moderate fibrosis (F < 3, n = 234) and significant-to-advanced fibrosis (F ≥ 3, n = 40).

Statistical analysis indicated that age, gender, and HBeAg positivity/negativity did not significantly differ between the two fibrosis groups (*p* > 0.05). Similarly, no significant correlation or association was observed between these demographic variables and the Knodell HAI when stratified at the ≤6 vs. >6 threshold. These findings suggest that in this cohort, clinical staging based on non-invasive indices is not confounded by age or gender.

### 3.7. Correlation Analysis of Clinical Indices and Histopathological Scores

The relationship between non-invasive indices (PNI and SII) and histopathological findings was evaluated using Spearman’s rank correlation coefficient (rho). A significant, albeit weak, negative correlation was observed between the PNI and Ishak fibrosis stages (r = −0.279, *p* = 0.02), suggesting that nutritional and immunological status decline as hepatic scarring progresses.

Conversely, SII showed a significant but weak positive correlation with fibrosis severity (r = 0.244, *p* = 0.01), suggesting an association with increased systemic inflammatory activity in advanced disease stages. Regarding necro-inflammatory activity, no statistically significant correlations were found between the Knodell HAI and either PNI or SII (*p* > 0.05), indicating that these indices may be more representative of chronic architectural changes (fibrosis) rather than acute necroinflammation in this specific cohort.

### 3.8. Diagnostic Performance of PNI and SII in Predicting Fibrosis Severity

Diagnostic accuracy was evaluated using ROC curve analysis across Ishak fibrosis stages. The ROC curves were revised to clearly present AUC values, optimal cut-off points, and the comparative performance of PNI and SII to enhance interpretability. Optimal cut-off values were determined using the Youden Index, and clinical performance was assessed through sensitivity, specificity, and predictive values (PPV/NPV). However, given the modest diagnostic performance, these cut-off values should not be considered clinically definitive. The relatively high PPV combined with moderate NPV suggests that these indices may be more useful for confirming advanced disease (“rule-in”) rather than reliably excluding it (“rule-out”), thereby limiting their applicability as screening tools.

**Figure 1:**
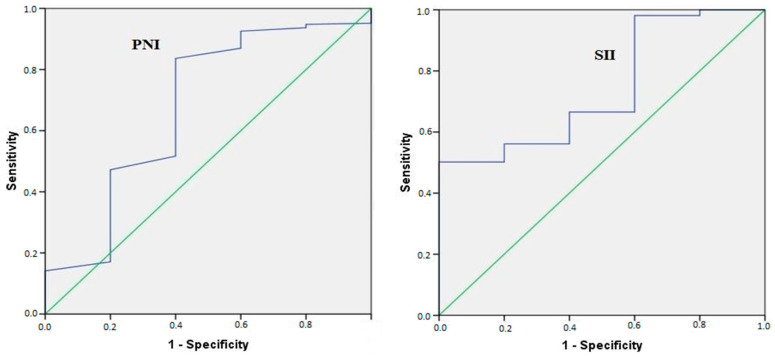
Receiver operating characteristic (ROC) curve analysis comparing the diagnostic performance of PNI and SII for advanced fibrosis (Ishak F0–F4 vs. F5–F6). AUC values are displayed to facilitate comparison between indices. Although SII demonstrated relatively higher performance than PNI, both indices showed only moderate discriminative ability.

- **Early-to-Moderate Fibrosis (≥F2 and ≥F3):** SII demonstrated statistically significant but limited discriminative ability for stage ≥F2 (AUC = 0.607, *p* = 0.013; cut-off: 347.10) and similarly modest performance for stage ≥F3 (AUC: 0.6–0.7). In contrast, PNI did not reach statistical significance for these stages, indicating limited utility in early-stage assessment. Importantly, AUC values around 0.60 reflect poor discrimination and approach random classification, underscoring the minimal clinical utility of these indices for detecting early-stage fibrosis.
- **Advanced Fibrosis and Cirrhosis (≥F4 and ≥F5):** Both indices showed statistically significant associations with advanced disease (*p* < 0.05) (Table 2, Figure 1). While PNI (≥48.75) exhibited high sensitivity (93.5%), its overall discriminative ability remained limited (AUC: 0.617). SII showed relatively better, but descriptively observed, performance, with AUC values of 0.735 for advanced fibrosis (≥F4) and 0.802 for cirrhosis (≥F5). However, the wide confidence intervals and relatively small subgroup sizes warrant cautious interpretation due to the potential risk of overfitting and unstable estimates.
- **Clinical Utility:** For advanced architectural changes, both indices achieved a PPV approaching 100%, indicating high reliability for “ruling in” disease. However, the moderate NPV (60–70%) suggests that while these markers may have a role as supportive confirmatory tools, they are insufficient when used alone and should be supplemented with imaging to effectively “rule out” early-stage fibrosis. Moreover, despite statistical significance, the overall diagnostic accuracy remains limited, particularly in early disease stages.
- **Diagnostic Performance of PNI and SII by HAI Stages:** The predictive capacity of PNI and SII for different levels of necro-inflammatory activity was evaluated using two HAI thresholds. Initially, patients were stratified into mild-to-minimal activity (HAI: 1–5) and moderate-to-severe activity (HAI: 6–18). While both indices reached statistical significance for predicting HAI ≥ 6 (*p* < 0.05), their AUC values ranged between 0.5 and 0.6. This indicates poor discriminatory performance, essentially close to chance level, and therefore minimal clinical utility.

These findings highlight that neither PNI nor SII provides reliable discrimination for lower levels of necro-inflammatory activity. Table 3 demonstrates the diagnostic performance for moderate necroinflammation (HAI ≥ 6).

**Table 2:** Diagnostic performance of clinical indices in predicting cirrhosis (Ishak F0–F4 vs. F5–F6).

	AUC (%95 GA)	*p*	Cut-Off	Sensitivity (%)	Specificity (%)	Youden Index	PPV (%)	NPV (%)
**PNI**	0.677 (0.405–0.949)	0.017 *	51.25	83.6	60.0	0.436	95.12	67.88
**SII**	0.802 (0.555–0.969)	0.002 *	416.16	50.2	100	0.444	96.25	60.77

* *p* < 0.05 denotes statistical significance. **AUC:** Area Under the Curve; **PNI:** Prognostic Nutritional Index; **SII:** Systemic Immune–Inflammation Index; **PPV**: Positive Predictive Value; **NPV**: Negative Predictive Value.

**Table 3:** Diagnostic performance for moderate necroinflammation (HAI ≥ 6).

	AUC (%95 GA)	*p*	Cut-Off	Sensitivity (%)	Specificity (%)	Youden Index	PPV (%)	NPV (%)
**PNI**	0.577 (0.485–0.629)	0.037 *	55.25	60.0	47.7	0.130	57.24	47.88
**SII**	0.579 (0.510–648)	0.035 *	424.26	54.2	44.5	0.170	54.22	48.96

* *p* < 0.05 denotes statistical significance. **AUC:** Area Under the Curve; **PNI:** Prognostic Nutritional Index; **SII:** Systemic Immune–Inflammation Index; **PPV:** Positive Predictive Value; **NPV:** Negative Predictive Value.

**Figure 2:**
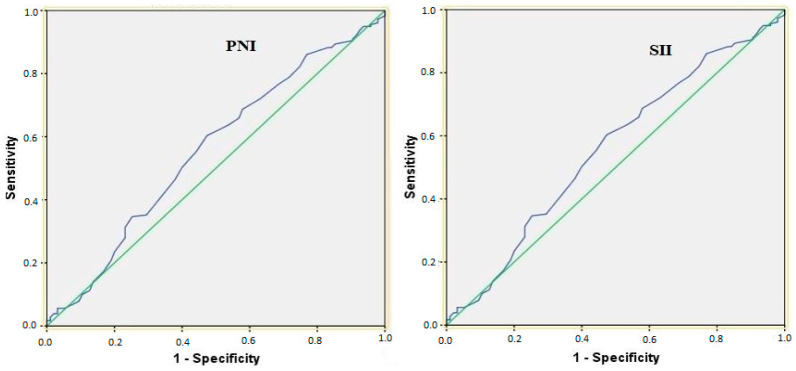
Receiver operating characteristic (ROC) curve analysis for predicting necro-inflammatory activity (HAI 1–5 vs. HAI 6–18). Both PNI and SII exhibited low AUC values (~0.5–0.6), indicating poor discrimination and limited clinical utility at this threshold.

The diagnostic accuracy of the model was further evaluated using Figure 2, which illustrates the receiver operating characteristic (ROC) curve analysis for predicting necro-inflammatory activity (HAI 1–5 vs. HAI 6–18).

- **Severe chronic hepatitis (HAI ≥ 12 vs. HAI < 12):** In a second analysis, the markers were evaluated for their ability to predict severe chronic hepatitis. In this context, the PNI failed to demonstrate significant predictive value (*p* = 0.059). In contrast, SII showed moderate diagnostic performance (AUC = 0.848, 95% CI: 0.704–0.992, *p* = 0.001). At a cut-off value of 408.26, it demonstrated a sensitivity of 84.6% and a PPV of 82.6%, although these findings should be interpreted cautiously.
- Given the very small sample size (n = 9), the observed performance should be considered exploratory and interpreted cautiously, as it may not be generalizable.
- **Severe Necroinflammation (HAI ≥ 12):** SII showed moderate performance for severe inflammatory activity (HAI ≥ 12; AUC = 0.848, *p* = 0.001), although results should be interpreted cautiously due to the small sample size. In contrast, PNI was not significant (*p* = 0.059).
- Although several findings reached statistical significance, the corresponding sensitivity, specificity, and AUC values remained modest, indicating limited clinical applicability. SII showed relatively better, but descriptively observed, performance compared to PNI.

**Figure 3:**
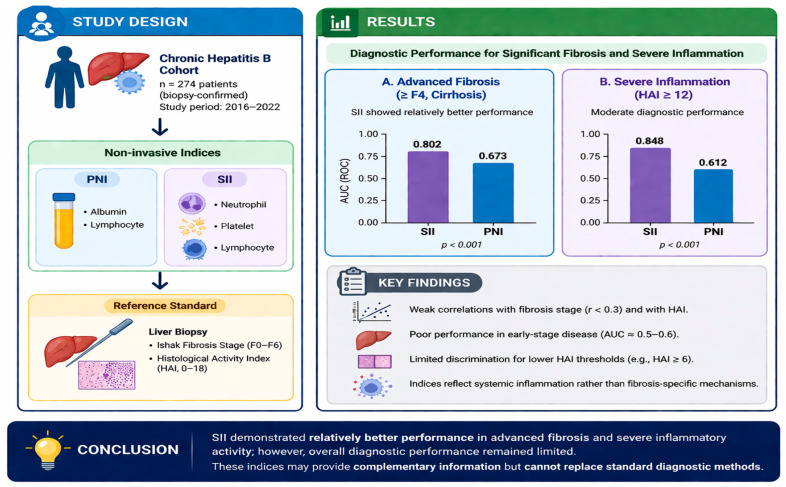
Diagnostic performance of PNI and SII in patients with chronic hepatitis.

Figure 3 summarizes the comparative performance of PNI and SII across fibrosis stages and HAI categories. SII showed relatively better performance than PNI in advanced fibrosis and severe inflammation; however, overall diagnostic accuracy remained limited. Both indices demonstrated poor performance in early-stage disease and should be considered complementary rather than standalone markers. This figure is intended for descriptive purposes only and does not represent a clinical decision-making algorithm.

## 4. Discussion

HBV remains a significant global health concern due to its potential to progress to cirrhosis, HCC, and acute liver failure requiring transplantation. Accurate assessment of liver fibrosis is essential for both prognosis and treatment decisions. Although liver biopsy remains the gold standard, its invasiveness, cost, and requirement for specialized expertise have led to increasing interest in non-invasive biochemical markers and scoring systems [9]. The present study demonstrates that systemic indices reflecting immune–inflammatory balance and nutritional status are associated with histopathological severity in CHB. Notably, SII showed relatively better—but still limited—diagnostic performance compared to PNI, particularly in identifying advanced fibrosis (≥F4) and severe necro-inflammatory activity (HAI ≥ 12). These findings should be considered exploratory due to the small sample size and interpreted with caution. However, this stage-dependent performance highlights an important limitation, especially in early-stage disease. These findings suggest that systemic inflammation may play a more prominent role than nutritional status in reflecting disease progression; however, both indices should be considered supportive rather than definitive biomarkers. While our results extend the current literature by linking these indices with biopsy-confirmed histopathological outcomes, the observed associations were modest and likely reflect overall systemic illness severity rather than fibrosis-specific pathophysiological mechanisms. Therefore, PNI and SII should not be considered robust or standalone biomarkers, but rather complementary indicators that may provide limited supportive information within a broader clinical context. As illustrated in Figure 3, the stage-dependent performance of these indices highlights their limited utility in early-stage disease and relatively better, but still constrained, performance in advanced stages.

Our analysis of demographic variables showed no statistically significant differences in gender when patients were stratified by fibrosis stage (≥F3, moderate-to-advanced) or HAI score (>6). Similarly, age and HBeAg status were not significantly associated with fibrosis stages or HAI scores. While some studies have reported a positive correlation between age and increasing fibrosis severity [10,11,12], others have found no such association [13,14], consistent with our findings. These discrepancies may be explained by differences in scoring systems (e.g., Metavir, Scheuer) or variations in sample size and patient characteristics, particularly the relatively small number of patients with moderate-to-advanced fibrosis in our cohort. In line with our results, previous studies have also indicated that gender and HBeAg status are not independent predictors of fibrosis severity [10,11,12,13,14,15].

Regarding biochemical parameters, platelet count and albumin levels showed statistically significant differences when patients were stratified using a cut-off of ≥F3 (bridging fibrosis), with both parameters decreasing as fibrosis progressed. However, their overall discriminative ability for fibrosis staging remained limited. In contrast, AST, ALT, lymphocyte, and neutrophil counts did not demonstrate significant discriminatory power for fibrosis severity. For necro-inflammatory activity (HAI > 6), AST, ALT, and albumin levels were significantly associated with higher activity, with increased transaminase levels and reduced albumin observed in patients with more severe inflammation. The limited discriminative ability observed for moderate necroinflammation (HAI ≥6) may reflect the inability of systemic inflammatory markers to capture localized intrahepatic inflammatory processes, particularly in earlier disease stages. Nevertheless, hematological parameters did not show significant or consistent associations with HAI scores, indicating limited utility in reflecting necro-inflammatory activity.

Comparisons with the literature reveal heterogeneous findings. Cai et al. [15] reported lower albumin and platelet levels, along with higher lymphocyte and neutrophil counts, in cirrhotic HBV patients. Similarly, Kekilli et al. [10], using the Metavir scoring system, identified associations with AST, ALT, albumin, platelet, and neutrophil levels. In contrast, Ben Ayed et al. [11] reported correlations primarily with AST and ALT, while Zhou et al. [12] found associations limited to albumin and platelet levels using the Scheuer scoring system. These discrepancies may be attributed to differences in scoring systems, sample sizes, and patient characteristics across studies. In our cohort, evaluated using the Ishak scoring system, albumin and platelet levels showed associations with fibrosis severity, consistent with previous findings [13]. However, these relationships should be interpreted cautiously, as they likely reflect underlying hepatic synthetic dysfunction rather than specific or independent indicators of fibrosis. In this context, the HAI score primarily reflects inflammatory activity associated with hepatocellular injury, whereas the Ishak score more closely represents cumulative structural changes and impaired synthetic capacity.

Correlation analysis revealed that PNI was negatively associated with liver fibrosis, while SII showed a positive association. However, these correlations were weak (r < 0.3) and of limited clinical significance, suggesting that neither index possesses the diagnostic strength required to serve as a standalone marker. ROC analysis further demonstrated that SII had limited discriminatory ability in early fibrosis stages (≥F2, ≥F3), with AUROC values around 0.60, indicating poor discrimination and minimal clinical utility. In contrast, SII showed relatively better but still moderate performance in advanced fibrosis (≥F4) and cirrhosis (≥F5), with AUROC values of 0.735 and 0.802, respectively. These modest associations may partly reflect residual confounding inherent to the retrospective design, as factors such as intercurrent illness, nutritional variability, and subclinical inflammatory conditions may influence systemic inflammatory markers. Accordingly, these indices should be interpreted with caution. Although SII appeared to perform better than PNI in certain subgroups, particularly in advanced disease, the magnitude of this difference was limited. Previous studies have highlighted SII and related inflammatory markers (e.g., NLR, PLR) as prognostic indicators in oncological settings, particularly in HCC [16,17,18,19,20,21,22,23]. Elevated SII has been associated with poorer overall survival and recurrence-free survival, especially in patients undergoing liver transplantation or surgical resection [20,21,22]. However, these findings primarily reflect systemic inflammatory status rather than disease-specific pathological changes. In the present study, SII showed moderate diagnostic performance for severe inflammatory activity (HAI ≥ 12; AUROC = 0.848), although the small sample size (n = 9) warrants cautious interpretation. Overall, both PNI and SII demonstrated limited diagnostic performance, particularly in early-stage fibrosis, and are not clinically applicable in this setting. Even in advanced disease, wide confidence intervals and small subgroup sizes raise concerns regarding potential overfitting and estimate instability. Taken together, these findings suggest that while these indices, particularly SII, may provide supportive information in advanced disease, they are insufficient for detecting early-stage liver damage and should be interpreted within a broader, multimodal clinical context.

The progression of malignant disease and overall patient survival are influenced by the host’s nutritional status [23,24,25]. The Prognostic Nutritional Index (PNI), derived from albumin and lymphocyte levels, reflects both nutritional and immunological conditions; in HCC, low albumin levels are associated with impaired hepatic function and poorer outcomes [26]. As systemic inflammation contributes to nutritional decline [27], combining inflammatory indices such as SII with PNI may provide a broader assessment of patient status. This combined approach has shown prognostic relevance in oncological settings by integrating inflammatory, immunological, and nutritional parameters [28]. In the present study, although direct comparisons between these indices and Ishak scores are limited in literature, their prognostic significance has been reported in HCC and acute HBV. Previous studies have demonstrated that low PNI levels are associated with increased mortality in acute hepatitis and reduced survival following HCC resection [15,29,30,31], while elevated SII levels have been linked to poorer outcomes in post-transplant HCC patients [21]. Similarly, combinations of inflammatory and nutritional indices (e.g., PNI, NLR, PLR) have been associated with prognosis in chronic liver disease and related conditions [32,33,34,35]. However, these findings should be interpreted cautiously, as most evidence derives from oncological or acute liver disease settings rather than chronic hepatitis B. Accordingly, while such indices may provide supportive information, their applicability to fibrosis assessment in CHB remains limited. In this context, our results suggest that inflammation-based scores, including SII and PNI, may have a complementary role in assessing disease severity, particularly in advanced stages, but are insufficient as standalone markers for monitoring fibrosis progression.

### 4.1. Study Strengths

This study has several notable strengths. First, it includes a relatively large, biopsy-confirmed CHB cohort with standardized histopathological evaluation using both Ishak fibrosis staging and Knodell HAI scoring systems, ensuring reliable outcome assessment. Second, the study incorporates two complementary systemic indices, SII and PNI, which reflect distinct yet biologically relevant aspects of disease progression, namely inflammation–immune balance and nutritional/hepatic synthetic function. Third, the analysis evaluates diagnostic performance across multiple clinically meaningful thresholds (≥F2, ≥F3, ≥F4, ≥F5; HAI ≥ 6 and ≥12), allowing for stage-specific interpretation rather than a single dichotomous classification. Finally, the findings suggest that routinely available laboratory parameters may provide supportive information for non-invasive assessment of disease severity in CHB.

### 4.2. Study Limitations

Several limitations should be acknowledged. The retrospective, single-center design may introduce selection bias and limit generalizability. In addition, the relatively small number of patients with advanced fibrosis and severe necroinflammation, particularly for HAI ≥ 12, may have affected the precision of subgroup analyses. Longitudinal changes in PNI and SII were not assessed, and external validation was not performed. Although statistically significant, the observed correlations were modest, indicating a limited ability of these indices to capture the complex pathophysiology of CHB when used alone. Moreover, PNI and SII are systemic rather than liver-specific indices, and their components are closely linked to processes observed in advanced liver disease. Therefore, the observed associations may reflect downstream effects of fibrosis—such as impaired synthetic function, portal hypertension, and systemic inflammation—rather than true predictive capability, raising the possibility of circular interpretation. Accordingly, these indices should be considered reflective markers of disease severity rather than independent predictors.

Residual confounding cannot be excluded due to the retrospective design, as factors such as intercurrent illness, nutritional variability, and subclinical inflammation may have influenced systemic markers. Additionally, the lack of adjustment for key confounders (e.g., antiviral treatment status, metabolic comorbidities, and inflammatory conditions) and the absence of multivariable analyses limit the ability to determine the independent contribution of PNI and SII. Finally, the lack of comparison with established non-invasive fibrosis markers (e.g., FIB-4, APRI, or elastography-based methods) limits the ability to determine the relative contribution and added value of these indices within the current diagnostic framework and further constrains the clinical interpretation of our findings.

### 4.3. Translational and Clinical Impact

The translational relevance of this study lies in its demonstration that routinely available, low-cost laboratory parameters may provide supportive information regarding histopathological disease severity. In clinical settings where access to biopsy or advanced imaging is limited, SII—alone or in combination with other modalities—may offer complementary value for patient monitoring. However, given the limited diagnostic performance observed, particularly in early-stage disease, these indices should be interpreted cautiously. The stage-dependent performance identified in this study suggests that their potential utility may be confined to advanced disease, where they could contribute to monitoring disease progression toward fibrosis or cirrhosis within a multimodal assessment framework.

### 4.4. Future Directions

Future research should focus on prospective validation in larger, multicenter cohorts and on evaluating the potential integration of SII and PNI into composite risk models that include imaging, elastography, and molecular biomarkers. In addition, longitudinal assessment of these indices may help to better characterize their role in disease progression and treatment response, although their clinical utility requires further clarification.

## 5. Conclusions

In this study, we evaluated the utility of PNI and SII in predicting biopsy-proven fibrosis and HAI scores in patients with chronic HBV. Our findings indicate that both indices have limited diagnostic performance, with only modest and potentially supportive value, particularly in advanced fibrosis and cirrhosis. Although SII showed relatively better performance than PNI, this difference was small and should be interpreted descriptively. Both indices demonstrated poor discriminative ability in early-stage disease, with AUC values approaching those expected from near-random classification. These findings highlight that statistical significance does not necessarily translate into clinical utility, particularly for potential screening markers. Accordingly, these indices should be interpreted with caution and considered complementary rather than standalone diagnostic tools. Importantly, PNI and SII represent non-specific systemic indices, and their associations with fibrosis likely reflect broader systemic inflammatory and nutritional alterations rather than direct involvement in fibrogenesis. Consistent with this, the weak correlations observed (r < 0.3) suggest limited biological relevance and indicate that these indices are more reflective of overall systemic status than fibrosis-specific mechanisms. While no non-invasive marker currently replaces liver biopsy, PNI and SII may provide supportive information when used alongside established methods such as imaging or elastography. However, in the absence of comparison with validated non-invasive tools, their clinical relevance remains difficult to fully contextualize. In settings where biopsy is unavailable or inconclusive, these indices may contribute to patient assessment; however, they cannot substitute standard diagnostic approaches. Overall, these findings support the use of PNI and SII within a multimodal assessment framework rather than as independent diagnostic markers. Future studies should focus on prospective validation and on clarifying their potential role in other liver diseases, including non-alcoholic fatty liver disease (NAFLD) and other viral hepatitis.

## Figures and Tables

**Table 1:** Descriptive statistics of clinical indices and histopathological scores (n = 274).

Parameter	Minimum	Maximum	Mean ± SD	Median
**PNI**	31.50	72.00	55.83 ± 5.34	56.00
**SII**	96.00	3198.25	494.37 ± 336.87	415.00
**Ishak Fibrosis Stage**	0	6	1.51 ± 1.10	1.00
**Knodell HAI Score**	1	15	5.94 ± 2.34	6.00

**PNI:** Prognostic Nutritional Index; **SII:** Systemic Immune–Inflammation Index; **HAI:** Histological Activity Index; **SD:** Standard Deviation.

## Data Availability

The data supporting the findings of this study are available from the corresponding author upon reasonable request.
